# The Effect of Metallic Nanoparticles Supplementation in Semen Extender on Post-thaw Quality and Fertilizing Ability of Egyptian Buffalo (*Bubalus bubalis*) Spermatozoa

**DOI:** 10.1007/s12011-024-04348-5

**Published:** 2024-09-10

**Authors:** Wael A. Khalil, Mohamed S. El-Rais, Mohamed M. Hegazy, Mahmoud A. E. Hassan, Ali A. El-Raghi, Mostafa M. El-Moghazy

**Affiliations:** 1 Department of Animal Production, Faculty of Agriculture, Mansoura University, Mansoura, 35516 Egypt; 2 Department of Animal, Poultry and Fish Production, Faculty of Agriculture, Damietta University, Damietta, 34517 Egypt; 3 Animal Production Research Institute, Agricultural Research Center, Ministry of Agriculture, Dokki, 12618 Giza, Egypt

**Keywords:** Metallic nanoparticle, Cryopreserved semen, Sperm characteristics, Kinematics, Apoptotic, Ultrastructure

## Abstract

Nanomaterials offer several promising prospects in the field of farm animal reproduction, encompassing a broad range of applications such as transgenesis and the precise delivery of substances to sperm cells, antimicrobial, antioxidants properties as well as their potent role in improving cryopreservation methods. The aim of the present study is to explore the effect of supplementing the semen extender with 10 µg/mL nano gold (Au-NPs10), 10 µg/mL nano silver (Ag-NPs10), 1 µg/mL nano selenium (Se-NPs1), and 100 µg/mL nano zinc oxide (ZnO-NPs100) on sperm characteristics and kinematics parameters, acrosome integrity, oxidative biomarkers, morphological and apoptosis-like changes of frozen-thawed buffalo bull sperm, and, ultimately, their fertilizing capacity. The results revealed that all aforementioned nano materials significantly improved viability, progressive motility, membrane integrity, acrosome integrity, and kinematic parameters as well as apoptosis-like changes of post-thawed buffalo bull sperm compared to the control (*p* < 0.05). No discernible effects were observed on sperm ultrastructure morphology measures as a response to the addition of these metallic nanoparticles to the extender. The values of caspase 3 significantly decreased by 64.22, 45.99, 75.59, and 49.39% in Au-NPs10, Ag-NPs10, Se-NPs1, and ZnO-NPs100 treated groups, respectively, compared to the control. The addition of 100 µg ZnO-NPs to the extender significantly decreased the total count of bacteria, fungi, and yeast compared to the control (*p* < 0.05). The AuNPs10 and SeNPs1 treated groups showed lower content of malondialdehyde, hydrogen peroxide, and nitric oxide concentrations and higher values of total antioxidant capacity of post-thawed extended semen (*p* < 0.05). Pregnancy rates increased by 17.5, 20, and 30% in buffalo cows inseminated with sperm treated with ZnO-NPs100, Se-NPs1, and Au-NPs10, respectively, compared to the control group. The present results indicate that the freezing extender supplemented with metallic nanoparticles can be an effective strategy to enhance the cryotolerance and fertility potential of buffalo bull sperm.

## Introduction

Water buffalo (*Bubalus bubalis*) is an imperative livestock species worldwide, providing several valuable resources, including milk, meat, draught power, and skin. However, water buffaloes face an exceptional set of challenges due to various factors such as long generation intervals, late sexual maturity, and silent heat, which hinder genetic improvement [1, 2]. Assisted reproductive technologies have been performed globally to promote the reproductive capacity of water buffaloes. These technologies include artificial insemination, in vitro maturation, in vitro fertilization, nuclear transfer, and cloning [2–4]. Semen cryopreservation has a crucial role in assisted reproductive technologies. The main objective of cryopreservation is to increase the post-thaw viability of spermatozoa and maintain their original quality parameters, including viability, motility, DNA integrity, acrosome integrity, and biological functions involved in fertilizing ability [5]. Despite being a well-established process in artificial insemination, semen cryopreservation still faces challenges, up to 50% of spermatozoa are unable to survive after the freeze–thaw process [2].

Cryopreservation can lead to cryo-injury in buffalo spermatozoa owing to several factors such as osmotic stress, cold shock, fluctuations in antioxidant protection systems, extreme reactive oxygen species (ROS) release, and intracellular ice crystal formation [6, 7]. During the freeze–thaw process, the levels of ROS significantly induce, resulting in an imbalance with the sperm’s antioxidant capacity and creating oxidative stress [8]. This oxidative stress leads to impaired fertilizing capacity due to sperm dysfunction. Water buffalo sperm is particularly sensitive to the events of cryo-storage owing to high levels of polyunsaturated fatty acids (PUFAs) in its plasma membrane, which make it more susceptible to oxidative stress compared to bovine sperm during cryopreservation or low-temperature storage [5]. These outcomes may explain the decreases in the fertilization ability of buffalo sperm following cryopreservation. While free radicals, such as superoxide anions, hydrogen peroxide, and nitric oxide, play primary roles in cell signaling, higher levels can disrupt sperm and contribute to male infertility [9]. Therefore, an effective tactic to enhance the cryo-resistance and fertilizing capacity of buffalo sperm is essential to improve sperm functionality and quality by strengthening the antioxidant defense system. This can be achieved by supplementing semen extenders with natural antioxidants [8].

In a study by Durfey et al. [10], conjugated metallic nanoparticles (NPs) were employed for molecular-based selection of boar spermatozoa, leading to notable improvements in motion characteristics, including a higher proportion of straightness and progressive spermatozoa. The use of antioxidants, such as nano-zinc oxide, is essential for enhancing sperm survival and reducing the generation of free radicals [11]. Incorporating 50 µg/mL zinc nanoparticles or 1 µg/mL selenium nanoparticles into a SHOTOR extender improved the ultrastructure and morphological characteristics of cryopreserved camel epididymal spermatozoa as response to the reduction of lipid peroxidation and apoptosis [12]. Supplementing a semen extender with 1.0 µg/mL selenium NPs has been observed to enhance post-thaw sperm quality and pregnancy rate in Holstein bulls due to the reduction of sperm damage, apoptosis, and lipid peroxidation [13]. Similarly, in rams, Nateq et al. [14] indicated that the use of selenium NPs at a dose of 1 µg/mL resulted in improvements in the viability index, motility, and membrane integrity of spermatozoa. Meanwhile, DNA fragmentation, acrosome defects, and lipid peroxidase concentrations were diminished.

It is important to note that gold and silver nanoparticles are considered nontoxic materials and possess various medical applications [15]. While it has been observed that these nanoparticles have the ability to penetrate the plasma membrane of human sperm and can be found inside the sperm nucleus, there is currently no evidence indicating their sperm toxicity [16]. The inclusion of 10 ppm green-synthesized gold nanoparticles in a Tris-based extender significantly affected buck semen freezing by preserving the integrity of the acrosome and sperm membrane after the thawing process. Furthermore, these NPs improved the redox status of the semen extender, causing the scavenging of ROS in buck semen extender [17]. In Wistar rats, Miresmaeili et al. [18] showed that the oral administration of 25 mg/kg silver NPs every 12 h during a spermatogenesis period of 48 days had a notable increase in the percentage of live sperms exhibiting acrosome reaction.

Therefore, investigating the use of a specialized semen-freezing extender containing metallic NPs holds promise for ensuring an optimal breeding platform specific to Egyptian buffalo. This study aimed to explore the effect of selenium, zinc oxide, silver, and gold NPs on various aspects of semen characteristics, kinematics parameters, acrosome exocytosis, oxidative biomarkers, caspase 3 activity, apoptosis-like changes, ultrastructure morphology of frozen-thawed buffalo sperm, and their fertilizing capacity. The outcomes of this research will involve the improvement of sperm cryopreservation techniques in Egyptian buffaloes.

## Materials and Methods

The current study was carried out at Mahalet Mussa Station, Sakha, Kafr El-Sheikh Governorate, Egypt, during the period from September 2023 to February 2024. All metallic nanoparticles utilized in the experiment were provided by Nano-gate Company, Cairo, Egypt. To verify the characteristics of the nanoparticles, a transmission electron microscope (TEM) with specific specifications (JEOL-JEM2100) and an acceleration voltage of 200kV. were used. The animal use and handling procedures were approved by the Animal care and use Committee (ACUC) at Mansoura University, Egypt, code number MU-ACUC (AGR.R.23.11.6).

### Animal Management and Semen Collection

Nine proven fertility buffalo bulls between the ages of 4 and 6 years were used in the current experiment. The bulls were subjected to standard management practices throughout the study. For a duration of 7 consecutive weeks, semen samples were obtained from each bull once per week (*n* = 63 total ejaculates) using artificial vaginas and carefully maintained at a temperature of 42–45 °C. To assess progressive motility, each ejaculate was assessed immediately after collection by two qualified researchers under a phase contrast microscope (100 ×). In the freezing experiment, only ejaculates with acceptable sperm concentration (≥ 8 × 10^8^/mL), abnormality (≤ 15%), progressive motility (≥ 75%), and viability (≥ 80%) were selected, pooled, and used for the study.

### Extender Preparation and Experimental Design

The extender used for diluting buffalo semen was prepared following the method outlined by Khalil et al. [19] by dissolving Tris 3.03 g, citric acid 1.68 g, fructose 1.25 g, glycerol 6.0 mL, and egg yolk 20 mL, in double-distilled water to obtain a final volume of 100 mL. Additionally, streptomycin (100 μg/mL) and penicillin (100 IU/mL) were also incorporated in the previous extender solution. Before cryoprotectants addition, the extender was adjusted for the osmolarity level to 280–300 mOsm (Micro-Osmometer, Loser Type 6, Löser Messtechnik, Berlin, Germany) and pH value of 6.8–6.9 (pH/mV Temperature Meter, Jenway 3510, Jenway, Staffordshire, UK). The extender was aliquoted into five 15 mL test tubes, and these test tubes were then stored in a water bath set at a temperature of 37 °C. The first test tube, which did not have any additional substances, was designated as the control, while extenders in the other test tubes were supplemented with 10 µg nano gold/mL (Au-NPs10), 10 µg nano silver/mL (Ag-NPs10), 1 µg nano selenium/mL (Se-NPs1), and 100 µg nano zinc oxide/mL (ZnO-NPs100). The semen was diluted with the extender at a ratio of 1:10 (sperm to extender), resulting in an initial dose of 80 × 10^6^ spermatozoa /mL in the diluted semen.

### Freezing and Thawing

Once the semen was diluted with the prepared extender, it was carefully shaken and then placed in a water bath at 37 °C. The diluted semen was subjected to an equilibration stage for 4 h at 5 °C. Thereafter, the equilibrated semen was packed into French straws, which had a volume capacity of 0.25 mL. These straws were obtained from IVM Technologies in Marseille, France. The packed straws were suspended at a height of 4 cm above the surface of liquid nitrogen vapors for a duration of 10 min, and then the straws were submerged in liquid nitrogen for cryopreservation for 1 month and then thawed at 37 °C for 30 s in a water bath for further assessments and analysis.

### Assessments of Semen Features

The sperm variables including sperm progressive motility, viability, abnormality, and membrane integrity were assessed after equilibration (5 °C for 4 h) immediately after thawing (at 37 °C for 30 s) and incubating in a CO_2_ incubator at 37 °C and 5% CO_2_ for 2 h [19]. The percentage of progressive sperm motility was examined using a phase-contrast microscope (DM 500, Leica, St. Gallen, Switzerland) equipped with a warm stage set at 37 °C and a magnification of 100 × . For the analysis, a 10 μL sample of the diluted semen was placed on a pre-warmed slide and covered with a coverslip. The analysis was performed by the same skilled investigator, blindly, and it was repeated three times for each sample [19]. Eosin/nigrosine stain method was used to assess the sperm viability as described by Moskovtsev and Librach [20]protocol. The solution of eosin (5%)/nigrosin (10%) was prepared by dissolving 0.9 g of sodium chloride and 5 g of eosin in distilled water (100 mL). The mixture was gently heated, and then 10.0 g of nigrosin was added. The solution was kept in dark conditions until use. For the experiment, samples of 10 μL from each group were incubated with 10 μL of the eosin/nigrosin solution for 2 min at a temperature of 25 °C. The samples were smeared onto hot glass slides for air-drying after incubation. Under a light microscope at 400 × magnification, at least 200 sperm cells were observed. During the microscope examination, the pink-stained sperm cells were considered dead, while the unstained sperm cells were considered viable. In the same regard, the percentages of sperm cells with abnormal head morphology (pear-shaped head, microcephalic head, loose head, round short head, double head) and tail morphology (terminally coiled tail, coiled tail, broken tail, double tail) were also recorded following the method of Samplaski et al. [21]. A hypo-osmotic swelling (HOST) test was used for assessing the membrane integrity (%) ratio, according to the method of Hassan et al. [5]. A total of 200 sperm cells were assessed for their ability to swell in HOST. Sperm cells with a coiled or swollen tail were considered to have an intact plasma membrane.

### Computer-Assisted Sperm Analysis

In the study of Dessouki et al. [22], the CASA (Computer-Assisted Sperm Analysis) sperm analyzer software, specifically Sperm Vision (Ref: 12,520/5000; Minitube Hauptstrae 41. 84,184 Tiefenbach, Germany), was applied to provide detailed information from live images of various sperm motion characteristics. Olympus computer-assisted microscope was used to capture these images. The microscope used in the study was the Olympus BX model (Hamburg, Germany) which was connected to a rapid scan digital camera capable of capturing images at a rate of 60 frames per second at 60 Hz under × 4 dark-field illumination. In each treatment, approximately 1500 spermatozoa were analyzed using CASA. The motion characteristics of the sperm were documented, and various parameters were measured, including distance straight line (DSL, μm); distance curved line (DCL, μm); distance average path (DAP, μm); velocity average path (VAP, μm/s); velocity curved line (VCL, μm/s); wobble (WOB = VAP/VCL); linearity (LIN = VSL/VCL); straightness (STR = VSL/VAP); amplitude of lateral head displacement (ALH, μm); and beat cross frequency (BCF, Hz).

### Assessment of Acrosome Integrity

To assess the rate of sperm acrosome integrity, the Giemsa Staining techniques were employed. In this method, 200 μL of frozen-thawed samples were placed into a plastic tube, and an equal volume of 0.2% trypan blue solution was incorporated. Thereafter, the tube was incubated at 37 °C in a water bath for 10 min. The sperm were extended with 2 mL of modified Oliphant and Brackett medium without albumin and subjected to centrifugation at 700 g for 6 min, following the protocol outlined by Brackett and Oliphant [23]. Then the supernatant was removed, and the spermatozoa pellet was re-suspended in 1–2 mL of the same medium and subjected to another round of centrifugation. The aforementioned process was repeated iteratively until the suspension reached either a pure or light blue color. Subsequently, 10–20 μL of the sperm suspension was placed onto a glass slide and smeared using a second glass slide. The slides were then rapidly dried on a 40 °C heating stage. To stain the sperms on the slides, they were exposed to a freshly prepared 10% Giemsa stock solution in distilled water for a duration of 1 h.

After the staining stage, the slides were rinsed with a stream of distilled water and left to air-dry. To assess the acrosome integrity using bright-field microscopy, approximately 100 sperm cells per slide were randomly selected and examined as follows:

- Viable sperms with an intact acrosome revealed a pink/purple staining in the acrosomal region; however, the post-acrosomal region showed white-stained. On the other side, viable sperm with exocytosed acrosomes exhibited a white post-acrosomal region, indicating true acrosome exocytosis.
- Non-viable sperms with an intact acrosome, the acrosomal region displayed a staining color varied between purple and dark pink. Moreover, the post-acrosomal region obtained a staining color ranging from dark blue to blue.
- Non-viable sperms with a reacted acrosome, the acrosomal region exhibited a staining of either white or gray colors, while the post-acrosomal region obtained a blue staining color, indicating a false acrosome.

### Oxidative Biomarkers Assays

The frozen-thawed semen samples were subjected to centrifugation at 4430 × g for a duration of 10 min. After centrifugation, the extender (solution) was separated from the samples and stored at a temperature of − 20 °C. The concentrations of MDA (malondialdehyde, MD 2529); TAC (total antioxidant capacity, TA 2513); and NO (nitric oxide, NO2533) were measured in extender according to the methods of [24, 25], at the wavelengths 532, 505, and 540 nm, respectively. The linearity indices for MDA, TAC, and NO were up to 100 nmol/mL, 2 mM/L, and 200 μmol/L, respectively. These indices indicate the maximum concentrations that could be accurately measured using the spectrophotometer (Spectro UV–Vis Auto, UV-2602; Culver City, CA, USA) employed in the study. The commercial kits were obtained from Biodiagnostic Company (Giza, Egypt) and were utilized following the manufacturer’s instructions.

### Assessment of Apoptosis Through Flow Cytometry

To evaluate apoptosis-like changes in frozen-thawed spermatozoa, a flow cytometry instrument was employed, and Annexin V staining was used [26]. The extended sperm from each group was centrifuged, and the isolated sperm cells were then suspended in 2 mL of binding buffer and thoroughly mixed. A 100 μL portion of the suspension was mixed with 5 μL of Annexin V (fluorescein isothiocyanate label) and 5 μL of PI (phycoerythrin label). The mixture was incubated in dark conditions for at least 15 min and, then, the samples were suspended in 200 μL of binding buffer. To assess the apoptotic status of the sperm, a flow cytometry examination was performed using an Accuri C6 Cytometer (BD Biosciences, San Jose, CA, USA) and software (Becton Dickinson) [27]. The percentages of negative or positive Annexin V (A − /A +), PI (PI − /PI +), and the double-positive cells were determined. According to the classification by Masters and Harrison [27], sperms were classified into four classes:

I. Viable cells: these are characterized by the absence of fluorescence signal and membrane dysfunction (A − /PI −).
II. Apoptotic sperm cells: viable sperm cells are labeled with Annexin V but without propidium iodide (A + /PI −).
III. Necrotic sperm cells: these are non-viable cells with complete membrane loss, labeled with PI but without Annexin V (A − /PI +).
IV. Necrotic sperm cells: non-viable sperm cells labeled with both PI and Annexin V as well as damaged permeable membranes (A + /PI +).

### Apoptotic Cascade

The primary executioner of the apoptotic cascade, caspase-3 (CASP3), was determined in post-thawed semen. Briefly, after washing the semen sample (100 μL, 1 × 10^6^/mL) with 2 mL of PBS/BSA, semen was centrifuged (2000 rpm for 5 min at 4 °C), then the supernatant was removed for exclusion of the particles of egg yolk, and the pellet was re-suspend in 100 μL of PBS. Thereafter, the semen sample was added to 10 μL of anti-active CASP3 (cat. No, 559,341) to incubate at dark for 30 min. After incubation, sperm were washed with 2 mL PBS/BSA and centrifuged (2000 rpm for 5 min at 4 °C), and then the supernatant was removed. Finally, sperm cells were re-suspended in 200 μL of 4% paraformaldehyde with PBS and fixed until picked up by flow cytometry [28, 29]. Flow cytometry was carried out by using Accuri C6 Cytometer (BD Biosciences, CA) supported with Accuri C6 software (Becton Dickinson).

#### Ultrastructure Assay of the Cryopreserved Spermatozoa

The ultra-structural modifications of buffalo-bull sperm cells were investigated using transmission electron microscopy (TEM) according to the protocol outlined by Khalil et al. [13], with some minor adjustments. Frozen-thawed semen was fixed in a solution of 2% glutaraldehyde in phosphate-buffered saline (PBS) for a period of 2–3 h and then subjected to three washes in PBS through centrifugation at 4 °C for 5 min each. Finally, the samples were incubated in a 1% osmium tetroxide solution at 4 °C for approximately 2 h. The fixed spermatozoa were dehydrated in acetone and then embedded in Epon resin. Ultrathin sections of the sperm samples were cut by the RMC ultra-microtome and stained with uranyl acetate and lead citrate. Randomly selected fields of the prepared sperm samples were examined using a transmission electron microscope (JEOL JEM-2100, Tokyo, Japan) equipped with an AMT Optronics CCD camera.

#### Total Bacterial, Fungi, and Yeast Count

Total bacterial counts of the examined semen were measured via the plate count method. Decimal dilutions were prepared and thoroughly mixed. Subsequently, 1 mL of the last three dilutions was transferred into Petri dishes (three replicated for each dilution). Thereafter, 15 mL of nutrient agar medium was added to each dish, left to hardness, and incubated for 72 h at 30 °C. The developed colonies were then counted [30]. The poured plate method was utilized for fungi examination. One milliliter of appropriate serial dilutions of the collected samples was inoculated into potato dextrose agar medium (PDA). Each inoculated sample was transferred into three sterile glass Petri dishes. Approximately 15 mL of PDA medium was poured into each plate after heating to 50 °C, then thoroughly mixed, and left to solidify. Three plates were incubated for 7 days at 25 °C. Ultimately the resulting fungal colonies in each plate were counted and recorded [31]. Malt extract agar was used to determine the yeast count according to the methods outlined by Atlas [32].

#### Fertility Rate

A total of 200 multiparous buffalo cows that displayed spontaneous estrus were randomly selected and divided into five equal groups (40 females/group). The control group constituted the first group, while the second, third, fourth, and fifth groups consisted of Au-NPs10, Ag-NPs10, Se-NPs1, and ZnO-NPs100, respectively. Individual’s buffalo cows were subjected to artificial insemination using frozen/thawed semen. A single AI technician conducted all the inseminations. At 60 days post artificial insemination, pregnancy status was determined using rectal palpation way.

#### Statistical Analysis

Data were edited in Microsoft Excel version 16 (Microsoft Corporation, Red-Mond, WA, USA). A Shapiro–Wilk test was performed in order to check for normality [33]. One-way ANOVA (PROC ANOVA; SAS Institute Inc., Madison, WI, USA, 2012) was used to examine the significant effects of the treatments, setting the level of statistical significance *α* = 0.05. The following mathematical model was applied to analyze all measurements, Yij = μ + TRTi + eij, where Yij = observations, μ = overall mean, TRT = effect of the different metallic nanoparticles (i, 1 to 5), and eij = random error. In case significant effects were detected, Tukey’s test was used to perform pairwise comparisons between means. Figures were fitted by the GraphPad Prism software 9.0 (Graph Pad, USA).

## Results

### Physicochemical Characterization of Nanoparticles

**Fig. 1:**
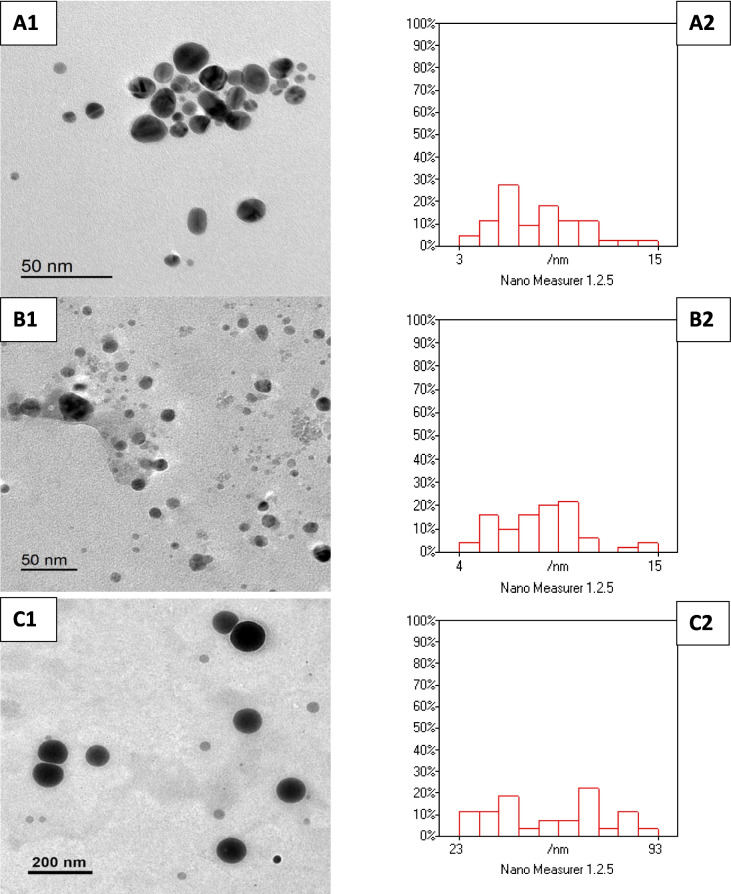

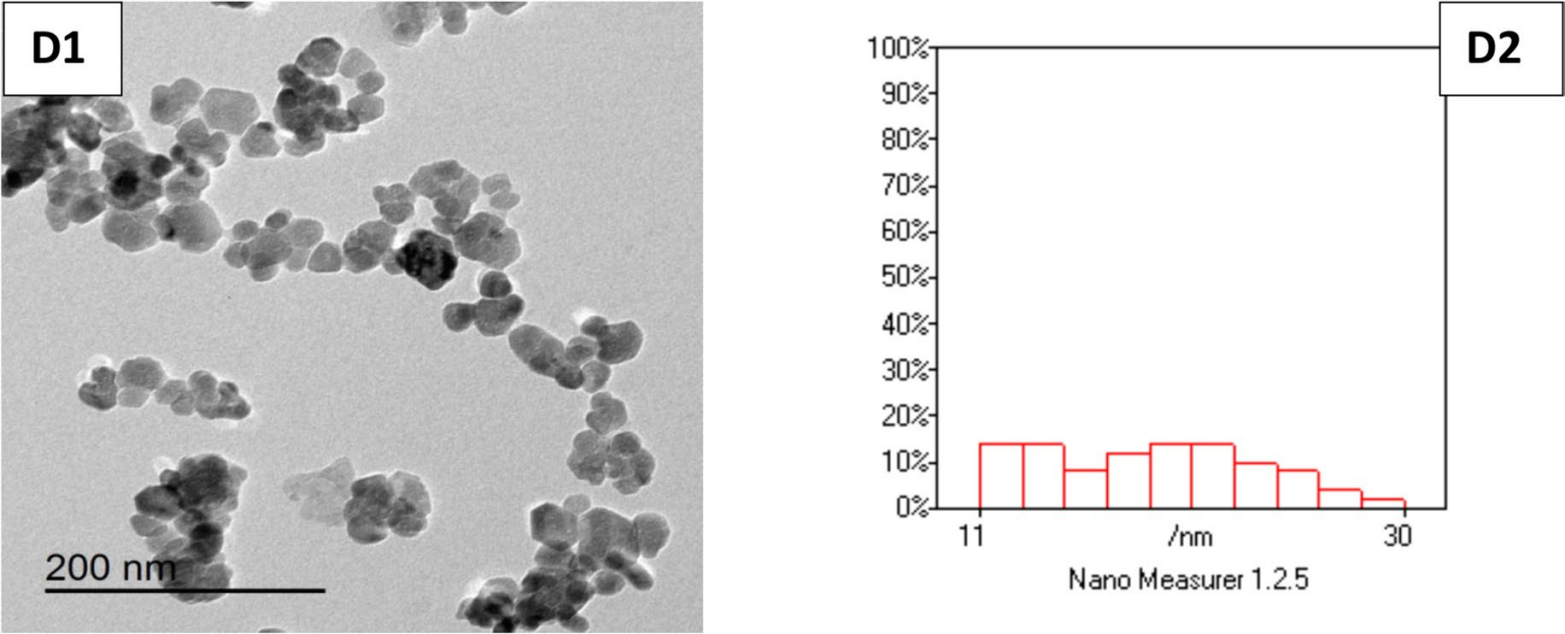
The characterization and morphology of different metallic nanoparticles including Au-NPs, Ag-NPs, Se-NPs1, and ZnO-NPs (**A1**–**A2**, **B1**–**B2**, **C1**–**C2**, and **D1**–**D2**) by transmission electron microscope (TEM). Histograms show a good distribution of NPs

The properties of metal nanoparticles were studied through the utilization of a transmission electron microscope (TEM; Fig. 1). The observations revealed that gold nanoparticles exhibita nano-scale size, ranging from 3 to 15 nm, and display a nearly spherical or spherical morphology. Similarly, silver nanoparticles also fall within the previous size range (3 to 15 nm), exhibiting a circular shape. For selenium nanoparticles, they exhibit a completely spherical shape and range varied from 32 to 93 nm. On the other hand, an irregular shape was detected with zinc oxide nanoparticles, tending towards a square morphology, with sizes ranging from 11 to 30 nm.

### Effects of Different Metallic Nanoparticles on Buffalo Bull Semen After Equilibration

As illustrated in Table 1, the supplementing freezing extender with different metal nanoparticles significantly improved sperm characteristics, including the progressive motility, livability, and membrane integrity of buffalo bull sperm after equilibration at 5 °C for 4 h compared to the control group (*p* < 0.0001), maximizing in both of the AuNPs10 and SeNPs1 treated groups. Non-significant differences were shown between the AgNPs10 and ZnONPs100 treated groups for all considered parameters (*p* > 0.05).

**Table 1:** Effect of supplementing freezing extender with different metallic nanoparticles on sperm characteristics (%) including progressive motility, livability, membrane integrity, and abnormality of buffalo bull sperm after equilibration at 5 °C for 4 h; the results were expressed as means ± SE, *n* = 9 replicates

Items	Treatments (TRTs)^1^					*p-*value
	Control	AuNPs10	AgNPs10	SeNPs1	ZnONPs100	
Progressive motility	72.78 ± 0.87^c^	82.78 ± 0.87^a^	78.33 ± 1.17^b^	83.33 ± 1.17^a^	78.89 ± 1.11^b^	< 0.0001
Livability	74.89 ± 1.28^c^	84.33 ± 1.05^a^	79.22 ± 1.07^b^	84.56 ± 1.30^a^	80.22 ± 1.17^b^	< 0.0001
Membrane integrity	75.00 ± 0.92^c^	83.22 ± 1.02^a^	78.78 ± 1.06^b^	84.33 ± 1.16^a^	79.44 ± 1.28^b^	< 0.0001
Abnormality	13.89 ± 0.56^a^	11.11 ± 0.51^b^	11.67 ± 0.55^b^	12.67 ± 0.53^a,b^	11.67 ± 0.47^b^	0.0051

^1^Different material nanoparticles including Au-NPs10 (nano gold, 10 µg/mL); Ag-NPs10 (nano silver, 10 µg/mL); Se-NPs1 (nano selenium, 1 µg/mL); and ZnO-NPs100 (nano zinc, 100 µg/mL) were added to the freezing extender. ^a–c^Values of the same row with different superscripts are significantly different (*p* < 0.05)

### Effects of Different Metallic Nanoparticles on Buffalo Bull Semen Immediately Post-thaw

The results of sperm characteristics of buffalo bull sperm after freezing are displayed in Table 2. The progressive motility, livability, and membrane integrity of buffalo bull sperm significantly improved by the metallic treatments (*p* < 0.0001), maximizing in the AuNPs10 treated group compared to the control and all other treated groups (*p* < 0.05). Meanwhile, there were non-significant differences between the AgNPs10 and ZnONPs100 treated groups for all aforementioned parameters (*p* > 0.05). The percentage of abnormal sperm was not affected significantly by the treatments (*p* = 0.1317).

**Table 2:** Effect of supplementing freezing extender with different metallic nanoparticles on sperm characteristics (%) including progressive motility, livability, membrane integrity, and abnormality of buffalo bull sperm after thawed at 37 °C for 30 s ; the results were expressed as means ± SE, *n* = 9 replicates

Items	Treatments (TRTs)^1^					*p*-value
	Control	AuNPs10	AgNPs10	SeNPs1	ZnONPs100	
Progressive motility	36.11 ± 0.73^d^	47.22 ± 1.47^a^	41.67 ± 0.83^c^	44.44 ± 0.56^b^	40.56 ± 0.56^c^	< 0.0001
Livability	37.89 ± 0.93^d^	47.89 ± 1.46^a^	43.56 ± 0.80^b,c^	45.67 ± 0.53^a,b^	42.33 ± 0.58^c^	< 0.0001
Membrane integrity	37.11 ± 0.71^d^	47.11 ± 1.05^a^	42.33 ± 0.84^b,c^	44.11 ± 0.51^b^	40.44 ± 0.55^c^	< 0.0001
Abnormality	17.00 ± 0.52	15.00 ± 0.67	15.78 ± 0.64	16.00 ± 0.47	15.22 ± 0.52	0.1317

^1^Different material nanoparticles including Au-NPs10 (nano gold, 10 µg/mL); Ag-NPs10 (nano silver, 10 µg/mL); Se-NPs1 (nano selenium, 1 µg/mL); and ZnO-NPs100 (nano zinc, 100 µg/mL) were added to the freezing extender. ^a–c^Values of the same row with different superscripts are significantly different (*p* < 0.05)

### Effects of Different Metallic Nanoparticles on Post-thawed Buffalo Bull Sperm Incubated at 37 °C and 5% CO_2_ for 2 h

As displayed in Table 3. The percentages of progressive motility, livability, and membrane integrity did not differ significantly between all treated groups (*p* > 0.05). However, these percentages were significantly higher than their counterparts in the control group (*p* < 0.05). Meanwhile, the treatments did not have discernible effects on the values of abnormal sperms (***p*** = 0.1123).

**Table 3:** Effect of supplementing freezing extender with different metallic nanoparticles on sperm characteristics (%) including progressive motility, livability, membrane integrity, and abnormality of post-thawed buffalo bull sperm incubated at 37 °C and 5% CO_2_ for 2 h; the results were expressed as means ± SE, *n* = 9 replicates

Items	Treatments (TRT)^1^					*p*-value
	Control	AuNPs10	AgNPs10	SeNPs1	ZnONPs100	
Progressive motility	30.56 ± 1.30^b^	38.89 ± 0.73^a^	37.78 ± 0.88^a^	39.44 ± 1.55^a^	36.11 ± 1.11^a^	< 0.0001
Livability	32.67 ± 1.33^b^	39.78 ± 0.94^a^	38.44 ± 1.08^a^	40.67 ± 1.31^a^	37.56 ± 0.96^a^	0.0001
Membrane integrity	31.11 ± 1.45^b^	38.56 ± 1.43^a^	37.78 ± 0.85^a^	39.00 ± 1.44^a^	35.22 ± 1.37^a^	0.0007
Abnormality	17.89 ± 0.39	16.11 ± 0.45	16.56 ± 0.60	17.11 ± 0.61	16.22 ± 0.49	0.1123

^1^Different material nanoparticles including Au-NPs10 (nano gold, 10 µg/mL); Ag-NPs10 (nano silver, 10 µg/mL); Se-NPs1 (nano selenium, 1 µg/mL); and ZnO-NPs100 (nano zinc, 100 µg/mL) were added to the freezing extender. ^a–c^Values of the same row with different superscripts are significantly different (*p* < 0.05)

### Effects of Different Metallic Nanoparticles on Kinematic Parameters in Post-thawed Buffalo Bull Sperms

In this study, all kinematic features (except the linearity and wobble) were significantly affected by the metallic treatments (*p*-values varied between 0.0370 to less than 0.0001). The values of TM, PM, DAP, DCL, VAP, VSL, and BCF were significantly enhanced in all treated groups compared to the control group (*p* < 0.05). The values of DSL were significantly higher in the SeNPs1 treated group than the control and the other treated groups (*p* < 0.05). Also, the treatment by SeNPs1 induced the values of VCL, being significantly higher than their counterparts in the control and the other treated groups (*p* < 0.05). Non-significant differences (*p* > 0.05) were observed between the control group and all examined groups except the ZnONPs100 for STR values. The values of ALH were significantly lower in the SeNPs1 treated group than the control and all treated groups (*p* < 0.05), non-significant differences were shown between the control group and both of the AuNPs10 and ZnONPs100 treated groups (*p* > 0.05) and the greatest values were obtained in the AgNPs10 treated group (Table 4).

**Table 4:** Effect of supplementing freezing extender with different metallic nanoparticles on kinematic parameters of post-thawed buffalo bull sperms; the results were expressed as means ± SE, *n* = 5 replicates

Items^1^	Treatments (TRTs)^2^					*p*-value
	Control	AuNPs10	AgNPs10	SeNPs1	ZnONPs100	
TM	58.12 ± 1.72^b^	69.50 ± 1.72^a^	67.97 ± 1.81^a^	69.54 ± 1.00^a^	65.33 ± 1.56^a^	0.0002
PM	30.60 ± 1.17^c^	49.53 ± 1.05^a^	45.06 ± 1.78^b^	45.06 ± 0.95^b^	43.37 ± 1.40^b^	< 0.0001
DAP	14.91 ± 0.38^d^	18.34 ± 0.36^b^	18.18 ± 0.63^b^	25.24 ± 0.57^a^	16.77 ± 0.10^c^	< 0.0001
DCL	22.35 ± 1.18^d^	28.85 ± 0.96^b^	28.64 ± 1.00^b^	37.67 ± 0.66^a^	25.34 ± 0.22^c^	< 0.0001
DSL	10.78 ± 0.11^b^	12.05 ± 0.26^b^	12.02 ± 0.29^b^	15.12 ± 1.10^a^	11.55 ± 0.13^b^	0.0001
VAP	38.79 ± 0.83^d^	46.62 ± 0.92^b^	46.31 ± 1.49^b^	55.18 ± 1.36^a^	42.46 ± 0.35^c^	< 0.0001
VCL	57.66 ± 3.05^c^	72.52 ± 2.47^b^	72.39 ± 2.77^b^	82.26 ± 1.95^a^	63.74 ± 0.90^c^	< 0.0001
VSL	24.87 ± 0.39^c^	31.07 ± 0.71^b^	31.13 ± 0.78^b^	35.69 ± 0.82^a^	29.58 ± 0.40^b^	< 0.0001
STR	63.60 ± 1.29^b^	66.20 ± 0.58^a,b^	66.80 ± 1.50^a,b^	64.20 ± 1.83^b^	69.20 ± 0.58^a^	0.0370
LIN	43.00 ± 2.26	42.20 ± 0.58	42.40 ± 0.81	42.80 ± 1.39	46.00 ± 0.63	0.2609
WOB	67.20 ± 2.18	64.00 ± 1.10	63.60 ± 1.36	66.60 ± 0.81	66.00 ± 0.71	0.2624
ALH	3.78 ± 0.07^b^	4.02 ± 0.06^a,b^	4.22 ± 0.07^a^	2.82 ± 0.24^c^	4.05 ± 0.13^a,b^	< 0.0001
BCF	14.81 ± 0.59^c^	18.69 ± 0.11^b^	18.55 ± 0.27^b^	24.34 ± 1.38^a^	18.26 ± 0.57^b^	< 0.0001

^1^*TM*, total motility (%); *PM*, progressive motility (%); *DAP*, distance average path (µm); *DCL*, distance curved line (µm); *DSL*, distance straight line (µm); *VAP*, velocity average path (µm/s); *VCL*, velocity curved line (µm/s); *VSL*, velocity straight line (µm/s); *STR*, straightness (VSL/VAP; %); *LIN*, linearity (VSL/VCL; %); *WOB*, wobble (VAP/VCL; %); *ALH*, amplitude of lateral head displacement (µm); and *BCF*, beat cross frequency (Hz). ^2^Different material nanoparticles including Au-NPs10 (nano gold, 10 µg/mL); Ag-NPs10 (nano silver; 10 µg/mL); Se-NPs1 (nano selenium, 1 µg/mL); and ZnO-NPs100 (nano zinc, 100 µg/mL) were added to the freezing extender. ^a–c^Values of the same row with different superscripts are significantly different (*p* < 0.05)

### Effects of Different Metallic Nanoparticles on Acrosome Integrity in Post-thawed Buffalo Bull Sperms

With respect to the acrosome integrity, the percentages of live sperm with intact acrosome were significantly higher in both the AuNPs10 and SeNPs1 followed by both AgNPs10 and ZnONPs100 treated groups compared to the control groups (*p* < 0.05). Regarding live sperm with detached acrosome, non-significant differences (*p* > 0.05) were shown between the control group and all examined groups (except the ZnONPs100 treated group), showing the greatest values. Dead sperms with intact acrosome decreased significantly in all treated groups compared to the control (*p* < 0.05), minimizing in the SeNPs1 treated group. Non-significant differences were observed between the control, AgNPs10, and SeNPs1 treated groups for the percentage of dead sperm with detached acrosome, and the minimum values were established in the AuNPs10 and ZnONPs100 treated groups (Table 5).

**Table 5:** Effect of supplementing freezing extender with different nanoparticles on acrosome integrity of post-thawed buffalo bull sperms; the results were expressed as means ± SE, *n* = 5 replicates

Items	Treatments (TRT)^1^					*p*-value
	Control	AuNPs10	AgNPs10	SeNPs1	ZnONPs100	
Live sperm with intact acrosome	40.00 ± 1.00^c^	52.40 ± 1.21^a^	47.20 ± 0.58^b^	54.20 ± 1.98^a^	44.60 ± 1.03^b^	< 0.0001
Live sperm with detached acrosome	15.00 ± 1.18^b^	14.40 ± 0.93^b^	17.40 ± 1.12^a,b^	14.80 ± 0.97^b^	19.00 ± 0.71^a^	0.0152
Dead sperm with intact acrosome	39.00 ± 1.00^a^	29.20 ± 1.39^b^	29.80 ± 0.49^b^	25.60 ± 1.21^c^	32.00 ± 1.10^b^	< 0.0001
Dead sperm with detached acrosome	6.00 ± 0.45^a^	4.00 ± 1.32^c^	5.60 ± 0.51^a,b^	5.40 ± 0.51^a,b,c^	4.40 ± 0.51^b,c^	0.0314

^1^Different material nanoparticles including Au-NPs10 (nano gold, 10 µg/mL); Ag-NPs10 (nano silver, 10 µg/mL); Se-NPs1 (nano selenium, 1 µg/mL); and ZnO-NPs100 (nano zinc, 100 µg/mL) were added to the freezing extender. ^a–c^Values of the same row with different superscripts are significantly different (*p* < 0.05)

### Effects of Different Metallic Nanoparticles on Redox Status and Nitrosative Biomarker in Post-thawed Buffalo Bull Sperms

**Fig. 2:**
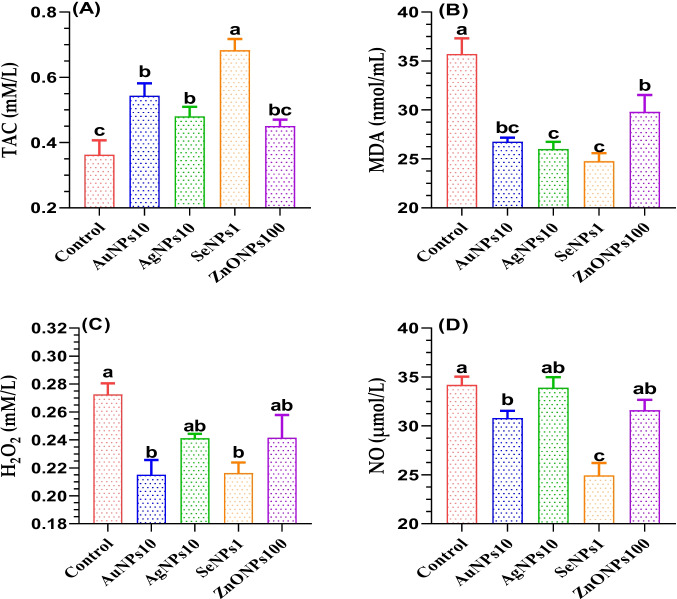
Effect of supplementing freezing extender with different nanoparticles on total antioxidant capacity (**A**), lipid peroxidase (**B**), hydrogen peroxide (**C**), and nitric oxide (**D**) in post-thawed buffalo bull semen. Au-NPs10 (nano gold, 10 µg/mL); Ag-NPs10 (nano silver, 10 µg/mL); Se-NPs1 (nano selenium, 1 µg/mL); and ZnO-NPs100 (nano zinc, 100 µg/mL). The results were expressed as means ± SE, *n* = 5 replicates

The results in Fig. 2 illustrate the effect of different metallic nanoparticles on total antioxidant capacity and oxidative and nitrosative biomarkers of post-thawed buffalo bull sperms. The SeNPs1-treated group showed the highest level of total antioxidant capacity and the lowest values of MDA and H_2_O_2_ compared to the control group (*p* < 0.05). The values of nitric oxide significantly decreased by 9.86 and 26.99% in AuNPs10 and SeNPs1 treated groups compared to the control (*p* < 0.05).

### Effects of Different Metallic Nanoparticles on Apoptosis-Like Changes and Caspase 3 of Post-thawed Buffalo Bull Sperms

**Fig. 3:**
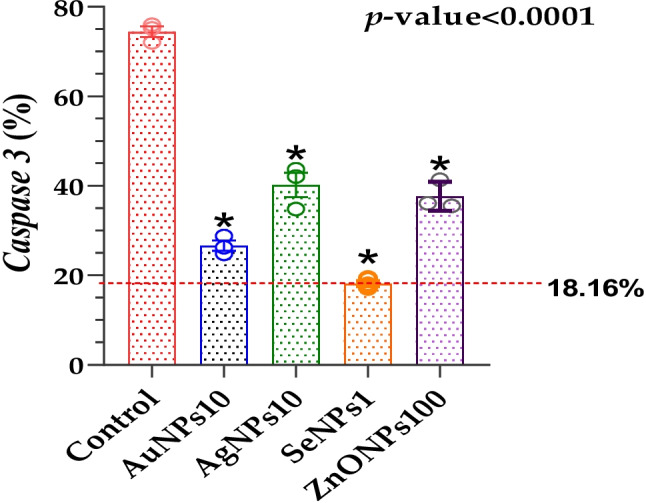
The values of caspase 3 in spermatozoa of buffalo received different nanoparticles including Au-NPs10 (nano gold, 10 µg/mL); Ag-NPs10 (nano silver, 10 µg/mL); Se-NPs1 (nano selenium, 1 µg/mL); and ZnO-NPs100 (nano zinc, 100 µg/mL). *p < 0.05: differ significantly with control. The results were expressed as means ± SE, *n* = 3 replicates

Herein, the supplementing freezing extender with different metal nanoparticles significantly induced viable sperm (*p* < 0.0001), maximizing in the SeNPs1 treated group followed by AgNPs10 > AuNPs10 > ZnONPs100. Early apoptotic sperms were significantly higher in the control group and decreased significantly in response to the treatments. The lowest level of necrotic sperms was observed in the SeNPs1 compared to the control and the other treated groups (*p* < 0.05; Table 6). As depicted in Fig. 3, the addition of different metal nanoparticles effectively decreased caspase 3 (as an apoptosis indicator) in the buffalo spermatozoa at cryopreservation. The lowest values were established in the Se-NPs1 treated group followed by Au-NPs10 < ZnO-NPs100 < Ag-NPs10.

**Table 6:** Effect of supplementing freezing extender with different nanoparticles on sperm apoptotic-like changes in post-thawed buffalo bull semen; the results were expressed as means ± SE, *n* = 3 replicates

Items	Treatments (TRT)^1^					*p*-value
	Control	AuNPs10	AgNPs10	SeNPs1	ZnONPs100	
Viable	36.33 ± 0.90^d^	51.07 ± 2.03^c^	53.03 ± 0.49^b^	65.43 ± 1.04^a^	50.97 ± 0.16^c^	< 0.0001
Early apoptotic	11.10 ± 0.05^a^	6.27 ± 0.18^c^	8.00 ± 0.06^b^	9.20 ± 0.58^b^	7.67 ± 0.03^c^	< 0.0001
Apoptotic	44.47 ± 0.84^a^	28.03 ± 0.35^b^	28.27 ± 0.64^b^	23.33 ± 1.27^c^	18.83 ± 0.21^d^	< 0.0001
Necrotic	8.10 ± 0.06^c^	14.63 ± 1.88^b^	10.70 ± 0.10^c^	2.03 ± 0.12^d^	22.53 ± 0.03^a^	< 0.0001

^1^Different material nanoparticles including Au-NPs10 (nano gold, 10 µg/mL); Ag-NPs10 (nano silver, 10 µg/mL); Se-NPs1 (nano selenium, 1 µg/mL); and ZnO-NPs100 (nano zinc, 100 µg/mL) were added to the freezing extender. ^a–d^Values of the same row with different superscripts are significantly different (*p* < 0.05)

### Sperm Ultrastructure

**Fig. 4:**
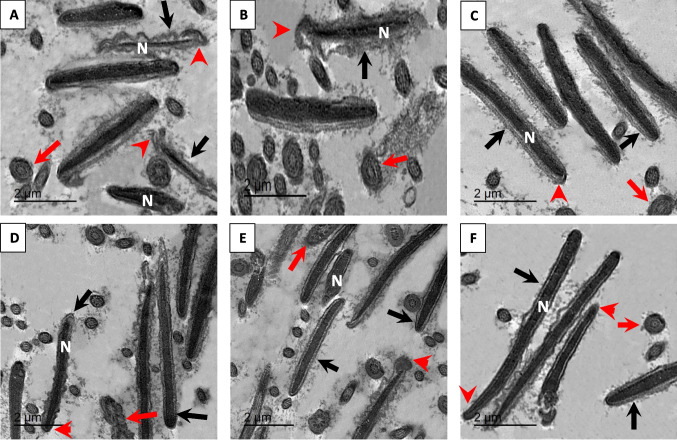
TEM photomicrographs of buffalo sperm head in the control (**A** and **B**) and experimental groups, including Au-NPs10 (nano gold, 10 µg/mL, **C**); Ag-NPs10 (nano silver, 10 µg/mL, D); Se-NPs1 (nano selenium, 1 µg/mL, **E**); and ZnO-NPs100 (nano zinc, 100 µg/mL, **F**).Plasma membrane (black arrow), acrosomal cap (arrow head), and mitochondria (red arrow)

After undergoing the freezing–thawing process, the cell membrane of the control group showed various changes in the plasma membrane (Fig. 4A and B), ranging from discontinuity to complete impairment. A ridge appeared at the tip of the acrosomal cap, which detached from the nuclear membrane, and the diffusion of acrosomal material. These alterations potentially resulted in the complete deterioration of the plasma membrane. Moreover, an abnormal nucleus with necrotic chromatin was clearly observable. Both the AU-NPS- and SE-NPS-treated groups (Fig. 4C and E, respectively) demonstrated numerous sperm cells with acrosomal caps, intact plasma membranes, and normal nuclei characterized by homogeneous chromatin condensation. However, the AG-NPS and ZNO-NPS treated groups (Fig. 4D and F, respectively) displayed moderate changes, indicating early damage stages in the acrosomal cap and plasma membrane.

#### Effects of Different Metallic Nanoparticles on *Bacteria*, Fungi, and Yeast Count

**Fig. 5:**
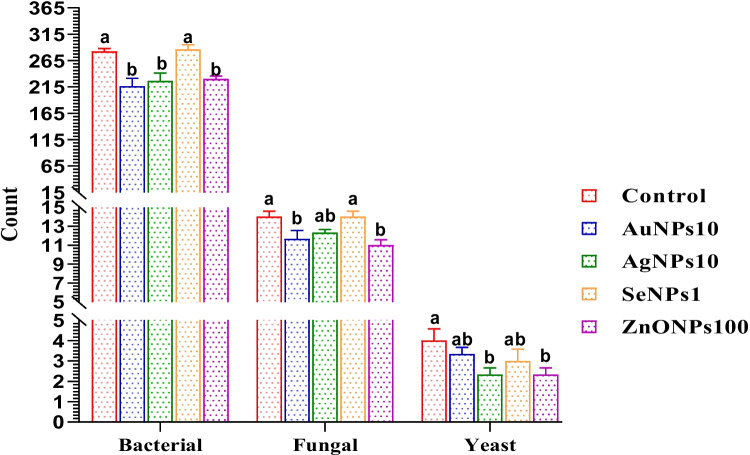
Bacterial, fungal, and yeast counts of buffalo bull semen received different nanoparticles including Au-NPs10 (nano gold, 10 µg/mL); Ag-NPs10 (nano silver, 10 µg/mL); Se-NPs1 (nano selenium, 1 µg/mL); and ZnO-NPs100 (nano zinc, 100 µg/mL). ^a–c^Values with different superscripts are significantly different (*p* < 0.05). The results were expressed as means ± SE, *n* = 3 replicates

The results indicate a notable diminish in the total count of bacteria, fungi, and yeast in the group treated with ZnONPs100 when compared to the control group (*p* < 0.05). However, there were no significant differences shown between the groups that received AuNPs10 and AgNPs10 (*p* > 0.05), as well as between the group treated with SeNPs1 and the control group as illustrated in Fig. 5.

#### Effects of Different Metallic Nanoparticles on Fertility Trial

**Fig. 6:**
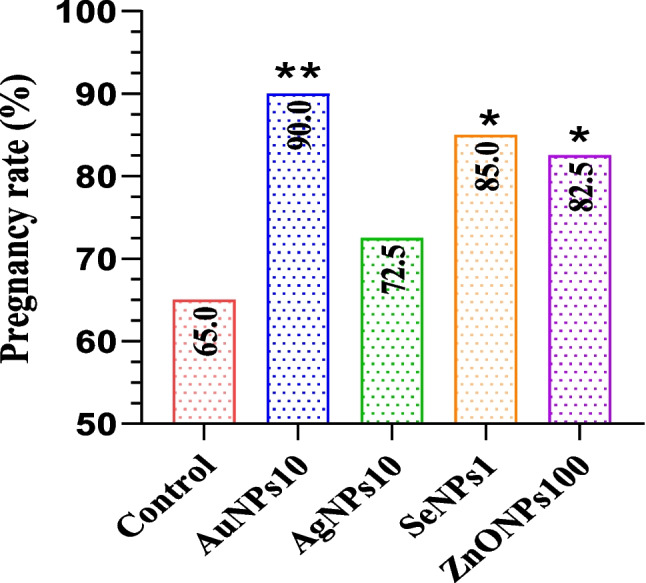
The effect of inseminated semen supplemented with different nanoparticles including Au-NPs10 (nano gold, 10 µg/mL); Ag-NPs10 (nano silver, 10 µg/mL); Se-NPs1 (nano selenium, 1 µg/mL); and ZnO-NPs100 (nano zinc, 100 µg/mL). *p < 0.05: differ significantly with control

As illustrated in Fig. 6, Buffalo cows inseminated with 10 µg Au-NPs had a higher pregnancy rate (*p* = 0.0074) followed by their counterparts received 1 µg Se-NPs (*p* = 0.038) and 100 µg ZnO-NPs (*p* = 0.049). Non-significant difference was observed between the pregnancy rate of Buffalo cows inseminated with10 µg Ag-NPs and those in the control group (*p* = 0.469).

## Discussion

Cryopreservation of buffalo semen has considerable detrimental impacts on various aspects of sperm quality, including membrane integrity, motion, fluidity, and permeability, which ultimately lead to impaired sperm viability, functionality, fertilizing ability, and mitochondrial functions. Buffalo sperm are particularly vulnerable to the generation of reactive oxygen species (ROS) induced by temperature changes and/or cryo-injuries due to their high polyunsaturated fatty acids content [34]. Elevated levels of ROS often lead to apoptosis, decreased cellular metabolism, and impaired acrosome reaction [35]. Therefore, it is crucial to enhance the freezing extenders with potent antioxidants such as metal nanoparticles to counteract the negative effects of ROS during cryopreservation.

In this study, the addition of gold nanoparticles (Au-NPs), silver nanoparticles (Ag-NPs), selenium nanoparticles (Se-NPs), or zinc oxide nanoparticles (ZnO-NPs) to the freezing extender of buffalo sperm yielded positive outcomes in terms of sperm characteristics, including progressive motility, viability, membrane integrity, and kinematic parameters as well as sperm ultrastructure. Moreover, they remarkably reduced acrosome damage and sperm apoptosis and enhanced the antioxidant capacity, showing a significant decrease in oxidative pathways such as H_2_O_2_ and MDA, and boosted the pregnancy rate in buffalo cows receiving the different metal nanoparticles-cryopreserved sperm. The present results were in general agreement with several former studies [13, 17, 36, 37] that showed noticeable effects of incorporating metallic nanoparticles on the morphological characteristics of semen after cryopreservation. Administration of Se-NPs (1 μg/mL) or Zn-NPs (50 μg/mL) in a SHOTOR extender has been observed to enhance the ultrastructure and morphological characteristics of cryopreserved camel epididymal spermatozoa [12]. Similarly, supplementation of a semen extender with Se-NPs (1.0 μg/mL) in Holstein bulls has been found to induce post-thaw sperm quality and thus conception rate by reducing apoptosis, sperm damage, and lipid peroxidation [13]. Moreover, in rams, several studies using Se-NPs (1 μg/mL) have shown improvements in progressive motility, membrane integrity, and viability index while reducing DNA fragmentation, acrosome defects, and malondialdehyde (MDA) levels as outlined by Hozyen et al. [38] and Nateq et al. [14]. The addition of green-synthesized gold nanoparticles at a concentration of 5 or 10 ppm to a Tris-based extender has been observed to enhance the redox balance of the semen extender leading to the scavenging of ROS [17]. Although Au-NPs and Ag-NPs can penetrate the plasma membrane and can be detected within the nucleus of human sperm [16], no evidence suggesting their toxicity to sperm cells [39].

Assessment of male fertility relies on the evaluation of sperm, which involves measuring various sperm quality parameters indicative of fertility. Nevertheless, semen evaluation has limitations, and it requires the implementation of rigorous quality control methods to interpret the obtained results [40]. Computer-assisted sperm analyzer (CASA) techniques enable the real-time examination of sperm kinetic, yielding accurate and rapid results [41]. They have promising predictive significance for assessing fertilization potential [42]. In this study, the Au-NPs10, Ag-NPs10, SE-NPs1, and ZnO-NPs100 treated groups showed significant improvements in the percentage of progressive motility and other motion parameters such as VSL, VCL, VAP, ALH, and BCF compared to the untreated group (*p* < 0.05). Consequently, metal nanoparticles can be considered natural substances for improving sperm functionality which may be attributed to the possibility that these materials induce the efficiency of ATP production, leading to improved energy levels and overall functionality of sperms. The other possible explanation for the observed increase in sperm motility and velocity characteristics could be attributed to the neutralizing of oxidative stress in treated groups compared to the control. The present results corresponded with the findings of Basioura et al. [43] who observed that the using of silver in semen extender significantly enhanced total and progressive motility, VCL, VAP, ALH, and BCF of boar sperms after thawing compared to the control group (*p* < 0.05). In rams, Nateq et al. [14] reported that the addition of 1 and 2 μg/mL nano-selenium to semen extender resulted in a significant increase in progressive motility, VAP, VSL, VCL, and ALH of sperm after thawing compared to the control group (*p* < 0.01). Also, Ozgur et al. [44] examined synthesized ZnO NPs (0.001 ppm) with *Cyprinus carpio* sperm samples and showed a significant increase in VSL and VCL compared to the control (*p* < 0.05), while VAP, BCF, and ALH did not affect significantly by the treatment (*p* > 0.05). Fouad and Ashour [45] reported significant enhancements in kinetic sperm parameters of Friesian-bull such as LIN, STR, and WOB after thawing as response to the addition of 0.5 mg SE/100 mL in freezing extender, which were in parallel with the significant improvements of motility parameters, vitality, acrosome integrity, and morphological abnormalities. Herein, the addition of ZnO NPs (100 µg/mL) to freezing media resulted in significant increase in STR by 8.81%, while both of LIN and WOB did not differ significantly (*p* > 0.05).

Mammalian sperm membranes have an abundance of polyunsaturated fatty acids, which make them more susceptible to lipid peroxidation; this process is the key mechanism of ROS-caused sperm damage, ultimately resulting in infertility [46]. Maintaining optimal sperm functionality and structure requires reducing the oxidative stress induced by the cryopreservation process [7]. Previous studies have highlighted the destructive impacts of elevated oxidative stress and reduced antioxidant capacity on various aspects of sperm function and structure, such as total and progressive motility, DNA integrity, plasma membrane integrity, and increase the percentage of apoptotic sperms [5, 19, 47, 48]. In this study, the inclusion of metallic nanoparticles into the extender of buffalo semen led to an increase in the values of TAC (*p* = 0.0002) and a decrease in the levels of lipid peroxidation (MDA; *p* < 0.0001) and H_2_O_2_ concentrations (*p* = 0.001) compared to the control group. In the cryopreservation trials on buffalo semen conducted by Khalil et al. [19], Khalil et al. [49], and Hozyen et al. [50], it has been observed that the using of nanoparticles can induce antioxidant indices after the freeze-thawing process with beneficial impacts on sperm functionality, what in consist with the present results. Similar results were also observed when the bull semen was enriched with the ZnONPs, resulting in decreased levels of MDA and enhanced mitochondrial activity and acrosome membrane integrity [51]. Additionally, the reduced bull semen MDA and increased TAC when using SeNPs in this study agreed with the findings in former studies [13, 52, 53] that showed significant improvements in antioxidant capacity and lipid peroxidation. Nateq et al. [14] reported that the supplementation of SeNPs (1 μg/mL) to ram semen extender significantly shrunken the levels of MDA and improved the oxidative capacity which could be attributed to the presence of selenium in glutathione peroxidase and Seleno-proteins [54]. In the same context, Dashtestani et al. [55] demonstrated that the utilizing of silver-gold-apoferritin nanozyme as a protective agent for human sperm against oxidative stress induced during the cryopreservation process increased the viability and motility of the sperm cells and also decreased the ROS, necrosis, and apoptosis compared to the control group (*p* < 0.05) due to the catalase activity of Ag-NPs [56] and Au-NPs [57].

Nitric oxide (NO) has the ability to reduce sperm motility and viability [58, 59]. This impact may be attributed to the potent role of NO as an inflammatory mediator in response to chronic or subclinical infection [60]. Additionally, NO has been noted to reduce the levels of cellular adenosine triphosphate (ATP) by diminishing ATP-generating enzymatic activities of the electron transport chain [61]. Since sperm motility depends on mitochondrial ATP production, a decrease in ATP production/sources leads to insufficient energy levels, which affects sperm motility. A significant negative correlation was observed between the higher levels of NO and impaired sperm function in various conditions, including asthenospermia, normospermia, leukocytoasthenospermia, leukocytospermia, and teratospermia among infertile individuals.

The acrosome reaction is a vital physiological process that sperms must undergo, aiming to successfully fertilize an oocyte. However, cryopreservation-induced spontaneous acrosome damage has been noted to decrease fertility rates [62]. Additionally, lipid changes due to oxidative stress are a key factor in inducing the percentage of sperm cells with an exocytosed acrosome in infertile bulls [7]. Therefore, it is essential to preserve acrosome integrity during the cryopreservation process by incorporating natural molecules into the freezing medium. Herein, the AuNPs10, AgNPs10, SeNPs1, and ZnONPs100 treated groups showed a notable relative increase in the percentage of live sperms with intact acrosomes by 30, 17.5, 35, and 10%, respectively, compared to the control group (*p* < 0.05). The present results corresponded with the findings of Zhandi et al. [63] who reported the beneficial effects of ZnO supplementation in semen extender on the acrosome integrity of rooster sperms which can result in better freezability. Also, Miresmaeili et al. [18] reported a significant increase in the percentage of live sperms with acrosome reaction in Wistar rats received orally administrated by AgNPs at a concentration of 25 mg/kg every 12 h in one spermatogenesis period (48 days), being 120.24% compared to the control group (*p* < 0.05). In goats, the addition of green-synthesized gold nanoparticles at a concentration of 5 or 10 ppm to a Tris-based extender has been observed to improve the freezing of buck semen after thawing by maintaining the integrity of sperm membrane and acrosome [17]. Nateq et al. [14] showed a significant reduction in the damage of acrosome membrane and the levels of MDA in the seminal plasma of bucks as response to the addition of 1 μg nano selenium to freezing media.

Apoptosis measurement has been recognized as an index of semen quality [64]. Therefore, it is crucial to control the rate of apoptosis in sperm cells for fertility purposes. Several studies have proven that the addition of metallic NPs such as AuNPs, AgNPs, SeNPs, and ZnONPs into the freezing extender can decrease apoptotic sperm and enhance fertilizing capacity [16, 17, 38, 65] which could be attributed to the ability of these NPs to diminish the ROS generated during the freezing process [66]. Moreover, mitochondrial ROS exposure can trigger the apoptosis of sperm cells and result in DNA fragmentation [67, 68]. The observed elevation in mitochondrial membrane potential in all treated groups compared to the control is likely attributed to a decrease in intracellular ROS which can be attributed to the examined nanoparticles. In the same context, the apoptosis process is characterized by specific biochemical events, including caspase activation, the relocation of phosphatidylserine to the external plasma membrane, and fragmentation of DNA [66]. Caspase activity has been involved in several sperm-related issues such as immaturity, low concentrate, reduced motility [69], lower fertilization levels [70], and compromised plasma membrane integrity. In this study, at the apoptosis transcriptomic level, all metallic NPs provided protection to buffalo sperm by decreasing the activity of caspase 3 in spermatozoa during the freezing process.

Regarding sperm ultrastructure, several former studies have indicated that when sperm cells are exposed to cold shock during the cryopreservation process, they exhibit various modifications in their structure. These alterations have been detected through transmission electron microscopy (TEM), including acrosome damage, mitochondria dysfunction, and increased fluidity of the plasma membrane [5, 19, 49, 71, 72]. Additionally, these changes in sperm morphology can potentially induce the proportion of apoptotic sperm and fluctuations resembling necrosis during both cryo-storage and freeze–thaw procedures [71, 73]. Saadeldin et al. [39] reported that the inclusion of metallic nanoparticles in the freezing media can improve the quality of sperm and functionality of the cryopreserved sperm. In this study, we discovered that the supplementation of freezing media of buffalo semen with metallic nanoparticles (NPs) can provide significant protection against cryo-damage via supporting the integrity of the sperm membrane, motion characteristics, and overall functionality of the sperm.

Bacterial contamination in mammalian semen negatively affects both the quality and longevity of sperms, leading to reduced fertility. Antibiotics have been commonly used to counter bacterial growth, but the escalating resistance of bacteria to various antibiotics poses a significant challenge. Fortunately, there have been recent advances in nanoparticle technologies, resulting in the development of several NP formulations with powerful properties such as anti-inflammatory agents, antioxidants, and antimicrobials [74, 75]. In this regard, AgNPs with sizes ranging from 10 to 20 nm have demonstrated biocide impacts on fungi and bacteria microorganisms without toxicity to certain mammalian cells [76]. Also, AuNPs have been observed to interact with the membranes of bacterial cells resulting in destroying their integrity and playing an antimicrobial and sterilizing property, as noted by Chu and Chen [77]. Additionally, Hayden et al. [78] observed that AuNPs protected by 2 nm core cationic monolayers can successfully interact with the cell membranes of both gram-negative and gram-positive bacteria, leading to a unique aggregation pattern and the lysis bacterial cell, indicating that the cationic surface properties of AuNPs can be utilized as antibacterial agents. Similarly, ZnONPs have demonstrated antibacterial, anticancer, and antioxidant features. According to the findings of Shinde [79], it was proposed that ZnONPs have the ability to generate ROS. Additionally, the oxidation capacity of these molecules towards γ-L-Glutamyl-L-cysteinyl-glycine (GSH) oxidation stress was identified as the contributing factor to their antifungal and antibacterial properties. The present results further support these findings by showing that the AuNPs10 and AgNPs10 treated groups were significantly lower in the bacterial counts, while the counts of fungi and yeast were significantly lower in AuNPs10 and ZnONPs100 treated groups than the control group.

The outcomes from the in vivo trial indicated that buffalo cows inseminated with sperm treated with Au-NPs10, Se-NPs1, and ZnO-NPs100 achieved pregnancy rates of 90%, 85%, and 82.5%, respectively, compared to 65% in the control group. This confirms the potential role of these nanoparticles to improve the cryopreserved buffalo semen and suggests their potential use in developing new types of extenders that can protect against or alleviate the detrimental impacts of the cryopreservation process, particularly in buffalo which have a high content of polyunsaturated fatty acids (PUFA) in their sperm structure. Moreover, the observed improvement in pregnancy rates in buffalo cows is associated with reduced sperm apoptosis and enhanced sperm motility as well as minimized acrosomal damage achieved through the use of metallic nanoparticles.

## Conclusions

The present study reveals the positive impact of metallic nanoparticles on improving the quality of buffalo sperm during cryo-storage. To elaborate further, the supplementation of these nanoparticles in extended semen decreases the oxidative stress biomarkers (MDA and H_2_O_2_), and apoptotic status and enhances various post-thawed sperm kinematic parameters, viability, progressive motility, acrosome integrity, membrane integrity, and total antioxidant capacity as well as sperm ultrastructure. Moreover, buffalo cows inseminated with sperm treated with ZnONPs100, Se-NPs1, and Au-NPs10 achieved increases in pregnancy rates by 17.5, 20, and 30%, respectively, compared to the control group.

## Data Availability

All data included in this study were presented in the form of tables and figures.
